# Superplastic Deformation of Alumina Composites Reinforced with Carbon Nanofibers and with Graphene Oxide Sintered by SPS—Experimental Testing and Theoretical Interpretation

**DOI:** 10.3390/ma15041396

**Published:** 2022-02-14

**Authors:** Rafael Cano-Crespo, César Retamal, Miguel Lagos, Francisco Luis Cumbrera

**Affiliations:** 1 Departamento de Física de la Materia Condensada, Universidad de Sevilla, Apartado 1065, 41080 Sevilla, Spain; fcumbreras@us.es; 2 Facultad de Ingeniería, Universidad de Talca, Campus Los Niches, Camino a los Niches Km 1, Curico 3340000, Chile; ceretamal@utalca.cl (C.R.); mlagos@utalca.cl (M.L.)

**Keywords:** alumina, plasticity, superplasticity, micromechanical modeling, ductility

## Abstract

The superplastic behavior of alumina-based nanostructured ceramics (Al_2_O_3_) is an important issue in the world of materials. The main body of this paper is an analysis of the creep behavior of polycrystals, with grain boundary sliding as the main deformation mechanism at high temperatures. Concomitant accommodation of grain shapes to preserve spatial continuity has a comparatively small effect on the strain rate. The constitutive equations for small deformations, relating strain and strain rate, derived from two models for grain sliding, are compared with the experimental data with their respective uncertainties. The data follow from experiments on the plastic deformation of alumina composites reinforced, on the one hand by graphene oxide, and on the other hand by carbon nanofibers sintered by SPS. The results show good agreement between experiment and theory for these advanced ceramics, particularly for one of the assumed models. The values obtained of *ξ*^2^ for model A were in the interval 0.0002–0.1189, and for model B were in the interval 0.000001–0.0561. The values obtained of *R*^2^ for model A were in the interval 0.9122–0.9994, and for model B were in the interval 0.9586–0.9999. The threshold stress was between (3.05 · 10^−15^–25.68) MPa.

## 1. Introduction

Advanced ceramic materials such as alumina composites (c-Al_2_O_3_) have good properties, and due to this, have several applications in the industry. These kinds of new materials have excellent properties of high strength, low chemical reactivity and high thermal and electric insulating characteristics [1,2].

For grain sizes *d* < 2 µm and in the temperature range (1373 ≤ T ≤ 1573 K), alumina samples obey the following Equation (1), which is a phenomenological equation proposed by Dorn

(1) $$\dot{\varepsilon }=A\frac{{\left(\sigma -\sigma_{0}\right)}^{n}}{d^{p}}exp\left(-\frac{Q}{k_{B}T}\right)$$

where $\dot{\varepsilon }$ is the strain rate defined by $\dot{\varepsilon }=d\varepsilon /dt$; in other words, the variation of the deformation with time. *A* is a stress and temperature independent coefficient, σ is the applied stress, $\sigma_{0}$ is the threshold stress which indicates the minimum value of stress to start the process of deformation and d is the mean grain size. Q is the activation energy of the deformation mechanism, which is defined by Equation (2), and can be obtained by changing the temperature and maintaining constant the value of stress. $k_{B}\;$ is the Boltzmann constant, and T is the absolute temperature. n is the stress exponent defined by the Equation (3), which is calculated by changing the applied load and maintaining the temperature constant. p is the grain size exponent. The value of *n* varies strongly with the flow stress σ, and sometimes takes the value *n* = 2.

(2) $$Q=-k{\left[\partial \left(ln{\dot{\varepsilon }}_{ss}\right)/\partial \left(1/T\right)\right]}_{\sigma_{ss}}\approx -k\left(\frac{ln\frac{{\dot{\varepsilon }}_{1}}{{\dot{\varepsilon }}_{2}}}{\frac{1}{T_{1}}-\frac{1}{T_{2}}}\right)$$

(3) $$n={\left[\partial \left(ln{\dot{\varepsilon }}_{ss}\right)/\partial \left(ln\sigma_{ss}\right)\right]}_{T}=\left(ln\frac{{\dot{\varepsilon }}_{2}}{{\dot{\varepsilon }}_{1}}\right)/\left(ln\frac{\sigma_{ss2}}{\sigma_{ss1}}\right)$$

The processes of deformation at high temperatures are as follows: (i) the grains slide along the common grain boundaries; (ii) the deformation of the grains try to accommodate to prevent the opening of holes and maintain the equation continuity; and (iii) accommodation of grain shapes produced by several processes like diffusion, dislocations, etc. [3].

Several authors [4,5] published theories that provided equations of the form (1) with *n* = 2 and *σ*_0_ = 0. Gittus [6] and Kaibyshev [7] introduced finite values for *σ*_0_. The grain size exponent changes from *p* = 1 [8], *p* = 2 [4,5,6,7,9,10,11] and *p* = 3 [6,12]. Hence, they attribute to the class of processes (iii) the main role in controlling the strain rate.

There are other models in the literature. The theory of Ashby and Verrall [13] provides *n* = 1. Gómez-García et al. [14] presented a model that explains the transition between two regimes with *n* = 1 and *n* = 2. This regime change has been reported by Sakuma and Yoshida [15]. 

Other models explain the superplastic regime of deformation [16,17,18,19,20]. A quite different scheme assumes that the reshaping of small grains demands weaker forces than those causing the sliding of the grains along the boundaries. In contrast to most of the theories cited in the previous paragraphs, this attributes to processes of class (i) the main role in controlling the strain rate. In this scheme, the flow is the composition of the sliding of all the grain pairs, which are driven by the shear forces in the shared faces of each pair. The grains continuously accommodate their shapes, but the forces demanded by these processes are much weaker. The final equation is analytic, and exhibits the characteristic sigmoidal shape of the logarithmic plots of the experimental stress-strain data for superplastic regime of deformation. The agreement between the theoretical equation and the experiment for several metal materials with grains of several microns (1.0 < *d* < 10 µm) is notable [16,17,18,19,20]. A recent paper shows that the model also fits with high precision the data of Zapata-Solvas et al. on ceramic samples [21,22]. The theoretical approach just described has two variants, which hereafter we will call models A [16] and B [19].

In this work, we begin revisiting the experimental data of Zapata-Solvas et al. [21]. These authors worked with 4% mol yttria tetragonal zirconia polycrystals (4-YTZP) with grain sizes between 0.38–1.15 µm. The employed temperature was between 1350–1400 °C, and the strain rates ranged between 5 · 10^−7^ and 2 · 10^−4^ s^−1^. The stresses were between 10 and 300 MPa. The stress exponents varied with increasing stress from 3.5 to an asymptotic value of 2.0. There is a correlation between the stress exponent and the grain size: the larger the grain size, the smaller the stress exponent, with a negligible influence of the applied stress. A value of the activation energy equal to *Q* = 520 ± 70 kJ/mol was also obtained from these creep tests, and no dependence on grain size was observed. These creep data were already analyzed in light of model B, to show that this model produces a better fit than the one of model A. The main part of this article is composed of the best fit analysis of both models, with experimental data on the plastic flow of alumina composites reinforced by graphene oxide on one hand, and carbon nanofibers sintered by SPS by Cano-Crespo et al. [23] on the other. These authors performed creep experiments at temperatures ranging between 1200–1250 °C, and the values of the applied load were in the range 9–300 MPa. The stress exponents were between 1.5 and 2.0 in both composites, and the activation energy was approximately 600 kJ/mol. The main theoretical hypotheses are discussed in detail, and the derivation of the constitutive equations is also discussed. The quality of the theoretical fits given by models A and B are compared, and the obtained values for the physical constants inherent to the models are discussed. 

## 2. Background Theory

The plastic deformation of a polycrystalline solid is modeled as the flow of a continuous material composed of a random array of deformable polyhedron, which can slide over each other along shared surfaces [18,19,20]. Each polyhedron continuously modifies its shape to fit the neighbors and preserve the continuity of matter. This describes how materials with micrometric or submicrometric grain size (<10 µm) do deform, and can undergo large plastic deformation. The hypothesis of this theory is that deformation is controlled by tangential forces on the grain boundaries. The shear stress must exceed a threshold value *τ_C_* to begin to slide. This stress is very low to activate dislocations, given the nature of the grains [24].

Other authors [16,17], Monte Carlo computer simulations [25], research on bicrystals [26] and molecular dynamic simulations [27] all indicate that the grains start to slide when the stress is higher than a critical value. 

There can be two kinds of elastic instability between grains when the stress is higher than a threshold value. There can be compression due to some boundary imperfections like triple points, and in this case only shear exists. In the former case, the amplitude of the corrugation increases linearly with the difference between the applied stress and the threshold stress for this kind of elastic instability, starting from zero. In the second class of instability, the plate suddenly breaks into bands when the threshold stress is reached. The resultant distortion jumps from zero to a finite value, and then starts to increase linearly with the applied stress. The relative velocity between adjacent grains is proportional to the amplitude of the induced stress field. Two possibilities for the force law between the sliding grains can be presented:

(a) The relative velocity $\Delta \vec{v}$ between two sliding grains is proportional to the difference between the shear stress and the threshold stress $\tau_{C}$.
(b) The relative velocity $\Delta \vec{v}$ increases linearly with the shear stress, and jumps from zero to the proper value when it is higher than $\tau_{C}$.

These two options are called models A and B, respectively.

In the Lagos model (model A), the speed $\left|\Delta \vec{v}\right|$ of two grains is proportional to the shear stress $\tau =\sqrt{{\left(\sigma_{x{}^{\prime }z{}^{\prime }}\right)}^{2}+{\left(\sigma_{y{}^{\prime }z{}^{\prime }}\right)}^{2}}$ applied in the sliding plane. Here $\sigma_{i{}^{\prime }j{}^{\prime }}$ denotes the elements of the stress tensor in the reference system whose *x*′*y*′ plane is in the sliding plane, shared by the two grains. The force law on the grain scale then reads

(4) $$\Delta v_{i{}^{\prime }}=\{\begin{array}{c} K\sigma_{i{}^{\prime }z{}^{\prime }}\;\\ 0\;\; \end{array}\begin{array}{c} \mathrm{if}\;\tau >\tau_{c},i{}^{\prime }=x{}^{\prime },\;y{}^{\prime }\\ \mathrm{if}\;\tau <\tau_{c},i{}^{\prime }=x{}^{\prime },\;y{}^{\prime } \end{array}$$

where *K* is a coefficient independent of the stresses. In this model the relative speed $\left|\Delta \vec{v}\right|$ of the grains jumps suddenly from zero to *Kτ_C_* when the shear *τ* stress reaches *τ_C_*. The Lagos-Retamal model (model B) [28] avoids such a discontinuity, and the relative speed increases proportionally to *τ*- *τ_C_*. The force law at grain scale for the two models can be written in a unified way as

(5) $$\Delta v_{i{}^{\prime }}=\{\begin{array}{c} K\sigma_{i{}^{\prime }z{}^{\prime }}\;\left(1-\frac{\;\alpha \tau_{c}}{\tau }\right)\\ 0\;\; \end{array}\begin{array}{c} \mathrm{if}\;\tau >\tau_{c},i{}^{\prime }=x{}^{\prime },\;y{}^{\prime }\\ \mathrm{if}\;\tau <\tau_{c},i{}^{\prime }=x{}^{\prime },\;y{}^{\prime } \end{array}$$

where

(6) $$\alpha =\{\begin{array}{c} 0:\mathrm{model}\;A\\ \;1:\mathrm{model}\;B\;\; \end{array}$$

The coefficient *K* only depends on the hydrostatic pressure

(7) $$p=-\frac{\left(\sigma_{x^{{}^{\prime }}x^{{}^{\prime }}}+\sigma_{y^{{}^{\prime }}y^{{}^{\prime }}}+\sigma_{z^{{}^{\prime }}z^{{}^{\prime }}}\right)}{3}$$

and temperature to be invariant to the surface orientation.

Equation (5) establishes a law which depends on the stresses, strains and strain rates fields. A change in the reference system must be made from the local reference system $x^{{}^{\prime }}y^{{}^{\prime }}z^{{}^{\prime }}$ to the absolute reference system xyz, and then promediate over all possible orientations of $x^{{}^{\prime }}y^{{}^{\prime }}z^{{}^{\prime }}$. The details of this procedure are in the literature [18,19,20,29]. The stress will be referred to as:

(8) $$\sigma_{xx}=\sigma_{yy}\equiv \;\sigma_{┴\;},\sigma_{zz}\equiv \;\sigma$$

The strain rate tensor can be obtained from

(9) $${\dot{\varepsilon }}_{ji}=\frac{1}{d}\langle \Delta v_{j}{}_{i}\rangle$$

where *d* is the mean grain size and the symbol ${\langle A\rangle }_{i}$ stands for the average of the values assumed by the magnitude *A* in a sequence of grains along the coordinate axis $x_{j}$. It obtained the following

(10) $$\dot{\varepsilon }=s\frac{K\tau_{c}}{\;2d\;}\left[cot\left(2\theta_{C}\right)+\alpha \left(2\theta_{C}-\frac{\pi }{2}\right)\right]$$

(11) $$\dot{p}=\frac{sBK\tau_{C}}{2d}\left[\frac{1-cos\left(2\theta_{C}\right)}{sen\left(2\theta_{C}\right)}-2\theta_{C}\left(1+\frac{2}{\pi sen\left(2\theta_{C}\right)}\right)-\frac{2cos\left(2\theta_{C}\right)}{\pi }+\frac{\pi }{2}\right]$$

where *ε* is the strain, s=± 1 is positive or negative for elongation or compression, *B* is the bulk modulus and $\theta_{C}$ is defined by:

(12) $$sen\left(2\theta_{C}\right)=\frac{4\tau_{C}}{3\left|\sigma +p\right|}$$

The stress $\sigma_{┴\;}$ changes when the sample is being deformed in the longitudinal direction *z*. It is essential to fully describe the state of the system at any instant, although it is better to employ the hydrostatic pressure.

(13) $$\;p=-\left(\sigma +2\sigma_{┴\;}\right)/3\;$$

Equations (10) and (11) govern the evolution of the deformation of a cylindrically symmetric solid along its axis.

If the solid has been annealed and has no residual internal stresses before subjecting it to uniaxial stress σ at time t=0, the internal pressure p(t) satisfies the initial condition $p\left(0\right)=-\frac{\sigma }{3}\;$, which is equivalent to $\sigma_{┴\;}\left(0\right)=0$, at any point inside the solid. In terms of the auxiliary variable $\theta_{C}$, this reads:

(14) $$sen\left(2\theta_{C}\right)=\frac{2\tau_{C}}{\sigma },\;at\;t=0$$

As long as the plastic deformation continues, ε, p and σ will evolve as dictated by Equations (10)–(12). The pressure p always increases, and this behavior has been postulated as the cause of solid materials having finite stress to fracture [29].

Information about *K*(*p*) is reported in [17,26]. A derivation can be found in [20]. The most important variations are in the chemical segregation of precipitation of a solid solution within the grain boundaries [30]. Pressure-dependent expression [16,17,20]

(15) $$\frac{K\left(p\right)}{4d}=C_{0}\frac{\Omega^{*}}{K_{B}T}\exp\left(-\frac{Q+\Omega^{*}p}{K_{B}T}\right)$$

was adopted to test whether the sensitivity $\Omega^{*}$ of the activation energy to the stress, appearing in Equation (15) through p, gives a good fit to the experiments. The function of internal pressure has been reported in olivine [31].

Combining Equations (10), (14) and (15) and substituting $p=-\frac{\sigma }{3}$, it follows that:

(16) ε˙=2C0Ω∗τcKBT[(σ2τc)2−1+α(arcsen(2τcσ)−π2 )]exp(−Q−Ω∗σ3KBT)

Recalling that α=0 for model A and α=1 for model B, the experimental data of a number of experiments were adjusted by Equation (16) to obtain the best values for the physical constants: $C_{0}$ (which depends on structure and grain size), the sensitivity $\Omega^{*}$ to stress of the activation energy, the critical shear stress $\tau_{c}$ for grain sliding and the activation energy Q.

## 3. Experimental Validation and Discussion

**Figure 1:**
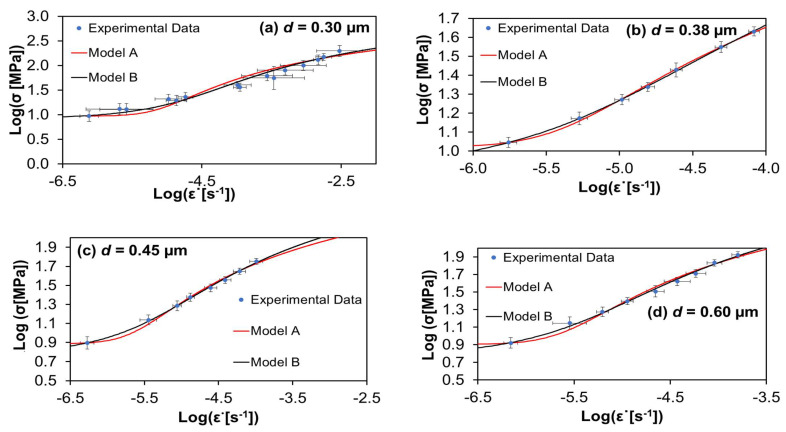

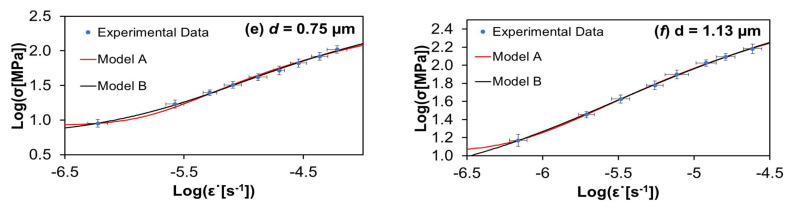
Stress σ versus strain rate $\dot{\varepsilon }$ data collected for creep tests of 4 mol% yttria tetragonal zirconia polycrystals at temperature of 1350 °C for six mean grain sizes. Circles are the experimental values of Zapata-Solvas et al. [21]. The continuous lines depict the best fit given by models A and B, respectively. The values for the statistical indices measuring the quality of the fits are given in Table 1 and Table 2.

The set of six graphs of Figure 1 shows the experimental points of ln σ vs. $ln\dot{\;\varepsilon }$ for the creep of 4-YTZP (4 mol% yttria tetragonal zirconia polycristal) measured by Zapata-Solvas et al. [21] for several grain sizes, together with the curves corresponding to the best fits given by models A and B. As it can be checked, all the experimental points with its bar errors adjust very well to the fitting curves.

Table 1 and Table 2 show the values of *ξ*^2^ and *R*^2^, indicative of the quality of the fitting of the experimental data by the two theoretical models. *ξ*^2^ has values for all the cases very close to zero, between 0.0001 and 0.1189. Furthermore, *R*^2^ has values between 0.9122 and 0.9996, which are very near to one. Both facts indicate a high quality of the fits. It can be observed there that, with the only exception of case *d* = 1.13 µm, in all other cases model B fits the data better than model A. In the mentioned case of *d* = 1.13 µm, the threshold value $\tau_{C}$ is higher than in the other cases due to the segregation of the yttria along the grain boundaries, and in Equation (5) for the model A we have that $\Delta v_{i{}^{\prime }}=K\sigma_{i{}^{\prime }z{}^{\prime }}$ and for the model B to high values of $\tau_{C}$ we have the same value of $\Delta v_{i{}^{\prime }}$, and this is the reason why in Figure 1f the fits of the experimental data for both models are similar. The values for the physical constants of the best fits are not shown, as they were published in a previous communication [22].

**Figure 2:**
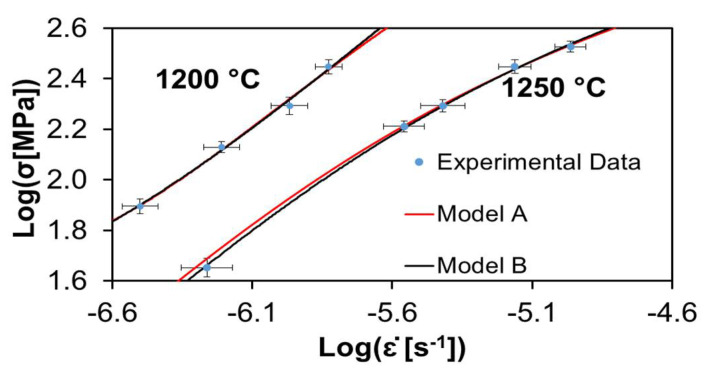
Creep data reported by Cano-Crespo et al. [23] for pure alumina (Al_2_O_3_) at two different temperatures. The circles represent experimental values. The continuous lines depict the best fit given by models A and B. The values for the statistical parameters that indicate the quality of the fits, and the corresponding physical parameters are given in Table 3, Table 4 and Table 5.

**Figure 3:**
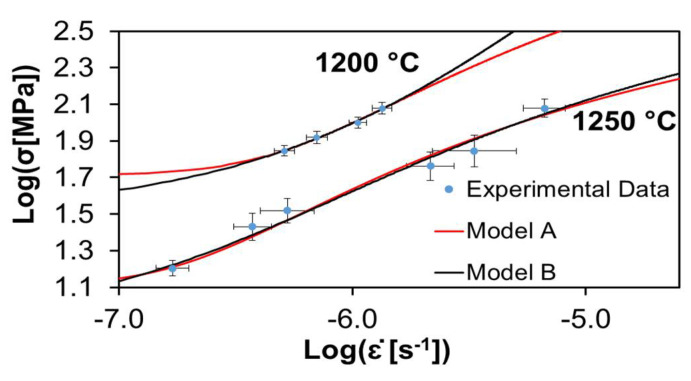
Creep data of alumina reinforced with graphene oxide measured by Cano-Crespo et al. [23] at two temperatures. The circles represent experimental values. The continuous lines depict the best fit given by models A and B. The values of the statistical parameters indicating the quality of the fits and the physical parameters are shown in Table 3, Table 4 and Table 5.

**Figure 4:**
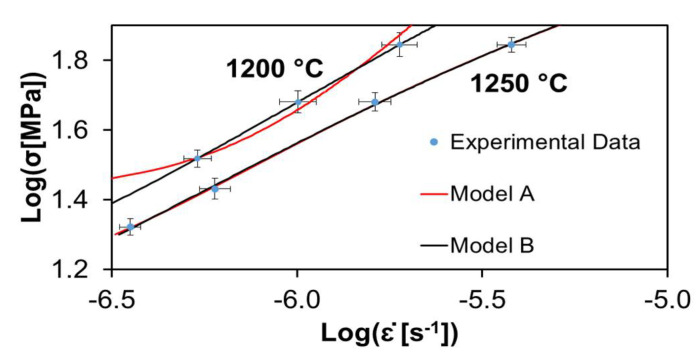
Creep data of alumina composite reinforced with carbon nanofibers sintered by SPS, measured by Cano-Crespo et al. [23] at two temperatures. The circles represent experimental values. The continuous lines depict the best fit given by models A and B. Values for the statistical parameters that indicate the quality of the fits and the corresponding physical parameters are given in Table 3, Table 4 and Table 5.

Figure 2, Figure 3 and Figure 4 show the experimental data of Cano-Crespo et al. [23] on the creep of composites of pure alumina (A), alumina-graphene oxide (A-GO) and alumina-carbon nanofibers (A-CNF) sintered by SPS, together with the corresponding fits of theoretical models A and B. Again, all the experimental values without exception are inside the fitting curves.

Table 3 shows the values of the statistical indexes *ξ*^2^ and *R*^2^, signaling the accuracy of the fits. *ξ*^2^ has values for all the cases very close to zero, between 0.000001 and 0.0191. Furthermore, *R*^2^ has values between 0.9501 and 0.9999, which are very near to one. Both facts indicate the high quality of the fits. It is also observed that model B fits the data better than model A for the two temperatures employed in the creep. Table 3 shows that for alumina-carbon nanofibers at a temperature of 1200 °C, model B gives a better fit than model A, but for a temperature of 1250 °C, model A fits better the experimental data.

Table 4 and Table 5 exhibit the values for the physical constants furnished by the best fit analysis. The activation energy was obtained directly from creep experiments [23], as only two temperatures were used. The critical stress $\tau_{c}$ in most cases is close to unity. For the same model and temperature, the relative values for $\tau_{c}$ satisfy the scheme A < A-GO > A-CNF. In most cases, for the same material and model, the temperature dependence of $\tau_{c}$ is such that $\tau_{c}$ (1250 °C) < $\tau_{c}$ (1200 °C), which is in agreement with what is expected, since it means that temperature reduces the threshold for flow stress, which can be understood because increasing the thermal molecular motions should weaken the bonds. Regarding the grain size dependent coefficient $C_{0}$, it can be seen that in general $C_{0}$ (1250 °C) < $C_{0}$ (1200 °C). Finally, for the parameter $\Omega^{*}$, it is generally true that $\Omega^{*}$(1250 °C) > $\Omega^{*}$(1200 °C). 

## 4. Conclusions

In this work, the data obtained from creep experiments on ceramic materials of composites of alumina reinforced with graphene oxide and with carbon nanofibers were fitted with two theoretical models already put forward in the literature, which we call models A and B. The values obtained of *ξ*^2^ for model A were in the interval 0.0002–0.1189, and for model B were in the interval 0.000001–0.0561. The values obtained of *R*^2^ for model A were in the interval 0.9122–0.9994, and for model B were in the interval 0.9586–0.9999. In most cases, model B gave a better fit. The best fit analysis for the alumina composites furnished the physical constants of the models, which were analyzed and commented on. The threshold stress was between (3.05 · 10^−15^–25.68) MPa. These findings were also tested for 4-YTZP materials, which confirm previous statements.

## Figures and Tables

**Table 1:** Quality of the fits of models A and B to the YTZP data of smaller grain sizes.

	d = 0.30 µm		d = 0.38 µm		d = 0.45 µm	
	Model A	Model B	Model A	Model B	Model A	Model B
*ξ* ^2^	0.1189	0.0561	0.0007	0.0001	0.0045	0.0013
*R* ^2^	0.9122	0.9586	0.9978	0.9996	0.9918	0.9977

**Table 2:** Quality of the fits of models A and B to the YTZP data of the higher grain sizes.

	d = 0.60 µm		d = 0.75 µm		d = 1.13 µm	
	Model A	Model B	Model A	Model B	Model A	Model B
*ξ* ^2^	0.0091	0.0033	0.0026	0.0007	0.0002	0.0003
*R* ^2^	0.9844	0.9943	0.9937	0.9983	0.9994	0.9990

**Table 3:** Values of the fitting parameters *ξ^2^* and *R^2^* for models A and B to the alumina (A), alumina-graphene oxide (AGO) and alumina-carbon nanofibers (A-CNF) creep data.

	1200 °C A		1250 °C A		1200 °C A-GO		1250 °C A-GO		1200 °C A-CNF		1250 °C A-CNF	
	A	B	A	B	A	B	A	B	A	B	A	B
*ξ^2^*	0.0008	0.0007	0.0012	0.0005	0.0003	0.0003	0.0191	0.0149	0.0022	0.000001	0.0005	0.0007
*R^2^*	0.9893	0.9913	0.9950	0.9979	0.9887	0.9909	0.9501	0.9611	0.9600	0.9999	0.9978	0.9965

**Table 4:** Values of the physical constants *Q* (J) and *C*_0_ (s^−1^) for models A and B to the alumina (A), alumina-graphene oxide (AGO) and alumina-carbon nanofibers (A-CNF) creep data.

	1200 °C A		1250 °C A		1200 °C A-GO		1250 °C A-GO		1200 °C A-CNF		1250 °C A-CNF	
	A	B	A	B	A	B	A	B	A	B	A	B
*Q* (J)	9.5 · 10^−19^	9.5 · 10^−19^	9.5 · 10^−19^	9.5 · 10^−19^	1.0 · 10^−18^	1.0 · 10^−18^	1.0 · 10^−18^	1.0 · 10^−18^	9.9 · 10^−19^	9.9 · 10^−19^	9.9 · 10^−19^	9.9 · 10^−19^
*C*_0_ (s^−1^)	3.46 · 10^14^	2.61 · 10^15^	3.10 · 10^13^	3.69 · 10^13^	3.43 · 10^15^	6.24 · 10^19^	2.68 · 10^14^	5.34 · 10^14^	8.53 · 10^20^	1.63 · 10^15^	1.06 · 10^14^	1.47 · 10^14^

**Table 5:** Values of the physical constants *τ_c_* (MPa) and Ω^*^ (m^3^) for models A and B to the alumina (A), alumina-graphene oxide (AGO) and alumina-carbon nanofibers (A-CNF) creep data.

	1200 °C A		1250 °C A		1200 °C A-GO		1250 °C A-GO		1200 °C A-CNF		1250 °C A-CNF	
	A	B	A	B	A	B	A	B	A	B	A	B
*τ_c_* (MPa)	19.84	7.92	0.25	3.05 · 10^−15^	25.68	15.83	6.32	3.58	13.28	3.85	6.78	2.63
Ω^*^ (m^3^)	4.9 · 10^−23^	8.3 · 10^−24^	2.5 · 10^−22^	2.3 · 10^−22^	2.1 · 10^−22^	2.5 · 10^−26^	8.7 · 10^−22^	6.7 · 10^−22^	1.2 · 10^−27^	4.5 · 10^−22^	1.1 · 10^−21^	1.0 · 10^−21^

## Data Availability

I choose to exclude this statement because this study doesn’t report any data.
